# Immunoglobulin G4-related Parotitis

**DOI:** 10.31662/jmaj.2023-0138

**Published:** 2023-11-16

**Authors:** Moe Watanabe, Shunichiro Hanai, Ryosuke Ito, Daiki Nakagomi

**Affiliations:** 1 Department of Rheumatology, University of Yamanashi Hospital, Chuo, Japan

**Keywords:** differential diagnosis, glucocorticoid, immunoglobulin G4, malignant tumor, parotid gland, tissue biopsy

A 74-year-old Japanese woman developed bilateral parotid gland swelling (Figure 1a), which, on examination, were smooth and slightly firm, with no observed tenderness or erythema. Open surgical excision biopsy of the parotid gland under general anesthesia was performed by otolaryngologists, and the obtained specimen showed a lymphoplasmacytic infiltrate with immunoglobulin G4 (IgG4)-positive cells and fibrosis, with no findings of obliterative phlebitis or storiform fibrosis (Figure 1b and c). The serum IgG4 level was elevated at 5080 (reference, <135) ^(1)^ mg/dL. Increased uptake of ^18^F-fluorodeoxyglucose was observed in the bilateral parotid, submandibular, and lacrimal glands as well as the pancreas and lymph nodes. IgG4-related disease was diagnosed based on the clinical, radiological, and pathological findings after consultation with pathologists, and medication with oral prednisolone 20 mg/day immediately alleviated swelling of the bilateral parotid glands (Figure 1d).

**Figure 1.**
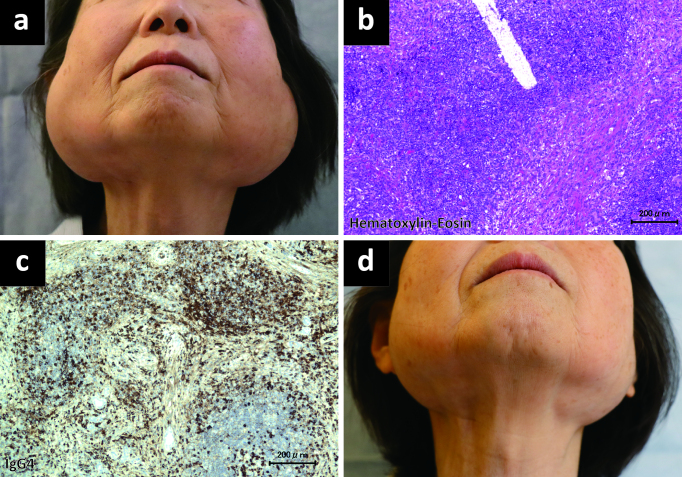
Changes in parotid gland swelling and histopathological findings over time. Bilateral parotid gland swelling before glucocorticoid treatment (a). On histopathological examination of the parotid gland biopsy specimen, a lymphoplasmacytic infiltrate with fibrosis (b) and immunoglobulin G4-positive cells (c) is seen. Swelling was alleviated after 10 days of glucocorticoid administration (d).

Many diagnoses for bilateral parotid gland enlargement include mumps, human immunodeficiency virus infection, tuberculosis, Sjögren’s syndrome, sarcoidosis, and Kimura disease ^(2)^. Of particular importance is the differentiation of IgG4-related disease from malignant tumors ^(3)^. Serum IgG4 concentrations are unreliable as a single diagnostic marker because multiple non-IgG4-related disease conditions are associated with elevated serum IgG4 levels ^(3)^; therefore, accurate diagnosis through tissue biopsy is needed for IgG4-related parotitis to differentiate it from infection, malignancy, or other conditions.

## Article Information

### Conflicts of Interest

None

### Acknowledgement

The authors would like to thank Dr. Aoi Kamijo from the Department of Otorhinolaryngology, Head and Neck Surgery for the parotid gland biopsy and Dr. Tomohiro Inoue and Naoki Oishi from the Department of Pathology for the pathological diagnosis.

### Author Contributions

Moe Watanabe and Shunichiro Hanai wrote the original draft, and Ryosuke Ito and Daiki Nakagomi reviewed and edited the final draft.

### Approval by Institutional Review Board (IRB)

Approval from the ethical board was not required by the authors’ institution because this is a single anonymous case report, with no intervention for research purposes.

### Informed Consent

Written informed consent was obtained from the patient by the corresponding author. The signed consent forms have been retained by the corresponding author. Patient details have been anonymized as much as possible.
